# Functional Balance between the Hemagglutinin and Neuraminidase of Influenza A(H1N1)pdm09 HA D222 Variants

**DOI:** 10.1371/journal.pone.0104009

**Published:** 2014-08-13

**Authors:** Jean-Sébastien Casalegno, Olivier Ferraris, Vanessa Escuret, Maude Bouscambert, Corinne Bergeron, Laetitia Linès, Thierry Excoffier, Martine Valette, Emilie Frobert, Sylvie Pillet, Bruno Pozzetto, Bruno Lina, Michèle Ottmann

**Affiliations:** 1 Université de Lyon, Université Lyon 1, Faculté de Médecine Lyon Est, Laboratoire de Virologie et Pathologie Humaine, EA 4610, Lyon, France; 2 Laboratory of Virology, National Influenza Centre (South of France), Hospices Civils de Lyon, Bât A3, Bron, France; 3 IRBA, Equipe Recherche Lyon, Lyon, France; 4 Université de Lyon, Université Lyon 1, Université Lyon 2, INSA de Lyon, École Centrale de Lyon, LIRIS UMR 5205 CNRS, Lyon, France; 5 Laboratoire de Bactériologie-virologie-hygiène, Hôpital Nord, CHU de Saint-Étienne, Saint-Étienne, France; National Institute for Viral Disease Control and Prevention, CDC, China, China

## Abstract

D222G/N substitutions in A(H1N1)pdm09 hemagglutinin may be associated with increased binding of viruses causing low respiratory tract infections and human pathogenesis. We assessed the impact of such substitutions on the balance between hemagglutinin binding and neuraminidase cleavage, viral growth and *in vivo* virulence.Seven viruses with differing polymorphisms at codon 222 (2 with D, 3 G, 1 N and 1 E) were isolated from patients and characterized with regards hemagglutinin binding affinity (Kd) to α-2,6 sialic acid (SAα-2,6) and SAα-2,3 and neuraminidase enzymatic properties (Km, Ki and Vmax). The hemagglutination assay was used to quantitatively assess the balance between hemagglutinin binding and neuraminidase cleavage. Viral growth properties were compared *in vitro* in MDCK-SIAT1 cells and *in vivo* in BALB/c mice. Compared with D222 variants, the binding affinity of G222 variants was greater for SAα-2,3 and lower for SAα-2,6, whereas that of both E222 and N222 variants was greater for both SAα-2,3 and SAα-2,6. Mean neuraminidase activity of D222 variants (16.0 nmol/h/10^6^) was higher than that of G222 (1.7 nmol/h/10^6^ viruses) and E/N222 variants (4.4 nmol/h/10^6^ viruses). The hemagglutination assay demonstrated a deviation from functional balance by E222 and N222 variants that displayed strong hemagglutinin binding but weak neuraminidase activity. This deviation impaired viral growth in MDCK-SIAT1 cells but not infectivity in mice. All strains but one exhibited low infectious dose in mice (MID50) and replicated to high titers in the lung; this D222 strain exhibited a ten-fold higher MID50 and replicated to low titers. Hemagglutinin-neuraminidase balance status had a greater impact on viral replication than hemagglutinin affinity strength, at least *in vitro*, thus emphasizing the importance of an optimal balance for influenza virus fitness. The mouse model is effective in assessing binding to SAα-2,3 but cannot differentiate SAα-2,3- from SAα-2,6- preference, nor estimate the hemagglutinin-neuraminidase balance in A(H1N1)pdm09 strains.

## Introduction

The influenza A(H1N1)pdm09 virus (H1N1pdm) emerged in March 2009 and caused the first influenza pandemic of the new millennium [1]. Despite appearing milder than expected, the severe cases and deaths that were reported worldwide far exceeded a classical seasonal flu. Worldwide, 18,500 deaths were reported but this figure was only based on laboratory-confirmed H1N1pdm cases [2]. One of the main features was the unusual occurrence of viral pneumonia amongst young and previously healthy individuals [3]. Preliminary epidemiological studies from Norway reported a higher frequency of the D222G mutation in hemagglutinin (HA, H1 numbering) of influenza viruses cultured from severe cases compared with mild cases [3]. This epidemiological association has since been confirmed in larger sample sizes in other countries using sequences obtained directly from respiratory samples [3], [4], [5], [6]. The D222G mutation in HA has only been detected sporadically and did not form a distinct phylogenetic cluster associated with a sustained chain of transmission [3], [6]. Further analyses have revealed that polymorphisms D, G, N, and E can be detected at position 222 in the HA protein of H1N1pdm viruses [7] but their impact on human pathogenicity remains to be determined. In mouse models, conflicting results have been reported, with some suggesting that D222G H1N1pdm viruses acquire an enhanced pathogenicity while others fail to display evidence of such an effect [8], [9].

The 222 HA residue is located near the receptor-binding site of the viral HA. Previous studies have indicated that the D222G substitution influenced the receptor binding specificity of the 1918 pandemic influenza A(H1N1) virus [10]. In human strains, it is D222 in HA that mostly favors binding to SAα-2,6; a receptor found in cells of the upper respiratory tract in humans. Binding to SAα-2,6 receptor is correlated with aerosol transmission. Whereas, in viruses emerging from the avian reservoir, it is G222 in HA that mostly favors binding to SAα-2,3. This receptor is found in avian cells but also in lower respiratory tract cells in humans (pneumocytes type II). Binding to SAα-2,3 receptor is correlated with viral pneumonia and pathogeny.

HA receptor binding properties are functionally dependent on the enzymatic properties of neuraminidase (NA). Indeed NA, like HA, engages glycan receptors on host cells, and their respective functional match is critical for viral fitness [11]. HA plays a role in viral entry into the target cell by recognizing the sialic acid receptor. The NA destroys the receptors by removing sialic acids. An optimal balance between these two functions and notably between HA binding affinity and NA sialidase activity is important for viral replication, as shown in culture [11] and *in vivo* [12].

In this study we assessed the properties of both HA and NA in relation to the *in vitro* and *in vivo* replication profiles of clinical H1N1pdm strains harboring a polymorphism at position 222. The purpose of our work was to assess if amino acid changes at position 222 only impacts the binding properties of the HA receptor or both the binding properties of HA and the HA-NA functional balance, and if this results in increased viral growth and virulence *in vitro* and *in vivo*.

## Materials and Methods

### Ethics statement for influenza strains

The influenza A(H1N1) viruses described in this study were collected from community and hospital settings. The samples were sent to the National Influenza Centre of the South as part of the national French influenza surveillance system. Patients provided written informed consent.

### Ethics statement for animal experiments

Animal care and experimental procedures were conducted according to French (décret 2001-131) and European (86/609/EEC directive) legislations. Protocols were approved by the Committee on Animal Experiments from the Université Lyon1 (Permit Number BH 2008–13). Animal welfare was checked daily and animal handling was performed after anesthesia with 5% isoflurane. Experiments were performed on six week-old female Balb/cByJ mice from Charles Rivers Laboratories.

### Sample collection and clinical data

H1N1pdm strains were isolated from nasopharyngeal swab samples obtained from seven patients admitted to the hospital between December 2009 and December 2011 and sent to National Influenza Centre of the South of France as part of the national influenza surveillance scheme. Seven influenza H1N1pdm viruses were selected based on their displaying polymorphisms of the HA protein at position 222 and having been isolated from patients presenting a broad range of clinical features (Table 1). Amongst the seven isolates, two displayed HA D222 (Lyon09D, Ste10D), three G222 (Reu10G, Ste09G, Lim10G), one N222 (Lyon10N) and one E222 (Lyon11E). As shown in Table 1, two patients with no comorbidities died from severe influenza, two patients recovered from severe influenza and three had mild symptoms. Isolates were obtained by culture of throat or nasal swabs on MDCK cells [13].

**Table 1:** Background information on the 2009 pandemic A H1N1 viruses.

Strain	Name	HA 222 AA	Collection date	Patient clinical symptom and outcome
A/Lyon/969/2009	Lyon09D	D	2009/06/18	Mild
A/StEtienne/1139/2010	Ste10D	D	2010/12/20	Dead
A/StEtienne/1691/2009	Ste09G	G	2009/09/14	Dead
A/Limoges/1159/2010	Lim10G	G	2010/12/22	Mild
A/Reunion/803/2010	Reu10G	G	2010/09/16	Mild
A/Lyon/52.016/2010	Lyon10N	N	2010/12/26	Febrile Purpura, Convulsions
A/Lyon/01.012/2011	Lyon11E	E	2011/01/01	Ressuscitation

### Virus, cells and in vitro replication kinetics

MDCK (ATCC, CCL34) cells were maintained in Ultra-MDCK serum free medium (Lonza) supplemented with 2 mM L-glutamine, 200 IU/ml penicillin (Lonza) and 200 IU/ml streptomycin (Lonza) at 37°C, 5% CO_2_. Influenza viruses were propagated in MDCK cells at 34°C in EMEM (Lonza) supplemented with 1 µg/ml trypsin (Sigma-Aldrich). MDCK-SIAT1 cells, which express higher levels of SAα-2,6 than parent MDCK cells [14], were maintained in DMEM (Lonza) supplemented with 2 mM L-glutamine, penicillin (200 IU/ml) and streptomycin (200 IU/ml, Lonza), 10% of heat inactivated fetal calf serum and 1 mg/ml G418 at 37°C, 5% CO_2_. MDCK-SIAT1 cells were infected at 34°C with viruses at a multiplicity of infection (MOI) of 0.001 TCID50/cell. To determine the virus yield, supernatants were harvested at fixed intervals post-infection and virus titers were assessed by endpoint titration in MDCK cells.

### DNA sequencing and analysis

Viral RNAs were extracted from the supernatant of infected MDCK cells using the QIAmp viral RNA mini kit (Qiagen) and reverse transcription was performed using Avian Myeloblastosis Virus Reverse Transcriptase (AMV RT, Promega). The cDNA products were amplified by high fidelity PCR (Phusion HotStart High-Fidelity DNA polymerase, Finnzymes) using primers specific for segments of the H1N1pdm virus (Table S2) [15]. The PCR products were purified using the MinElute purification kit (Qiagen) and sequenced (GATC). All viral sequences were analyzed and aligned using the ClustalW BioEdit software (version 7.1.83). Sequences are available on GenBank (Accession numbers Table S3).

### Antigenic characterization

Hyperimmune rabbit antisera and post-infection ferret sera were prepared as described previously [16]. Briefly, hemagglutination inhibition (HI) tests were performed as follows: rabbit sera to the reference strain A/Brisbane/59/2007 H1N1 and ferret sera to the influenza reference strains (A/NewJersey/8/1976 H1N1, A/Auckland/3/2009 H1N1, A/California/7/2009 H1N1pdm and A/Lyon/969/2009 H1N1pdm) were treated with receptor destroying enzyme (RDE) to remove non-specific inhibitors. Reference strains and isolates were standardized to four hemagglutination units and incubated with serial dilutions of sera. The HI titer (Table S1) was defined as the highest dilution of serum that still inhibited the agglutination of guinea pig erythrocytes (Charles Rivers Laboratories®).

### Hemagglutination assays

The hemagglutination assay (HA) was used to provide a quantitative assessment of the balance between HA binding and NA cleavage. HA titers were performed at two temperatures: 4°C and then 37°C. Viral supernatants (50 µl of a two-fold dilution in PBS) were incubated with 50 µl of 0.8% guinea pig erythrocytes in a 96 well plates for 75 minutes at 4°C. Viral elution was defined as the decrease in HA titer observed after incubation at 37°C for 2 additional hours. The HA assay was expressed as the mean of three experiments

### Kinetic analysis and end-point neuraminidase activities

The fluorometric NA activity assay and the half-maximal inhibitory concentration (IC50) determination for oseltamivir (kindly provided by Hoffmann-La Roche) and zanamivir (kindly provided by GSK) were performed twice for each isolate as previously described [17]. The Michaelis-Menten constant (*K*_m_) was measured from viral supernatants using the MUNANA substrate (Sigma-Aldrich) as previously described [18], [19]. The 4-methylumbelliferone fluorescence was measured using the FLUOstar OPTIMA fluorometer (BMG LABTECH) at 37°C. The initial velocities (Vi) were calculated for each substrate concentration and integrated in a non-linear Michaelis-Menten equation by the MARS program (BMG) for *K*_m_ and V_max_ determination. V_max_ reflects the activity of the enzyme, but this constant is also dependent on the virus quantity. NA activity was normalized by a quantitative assessment of the virus titer using endpoint titration in MDCK cells and expressed as V_max_ (nmol/h) per 10^6^ viruses. The NA activity was expressed as the mean of three experiments. The *K*_i_ (affinity of NA for an inhibitor) was measured across a concentration range from 0.01 nM to 10 nM. The substrate was used at 20 µM. The Vi was calculated for each inhibitor concentration and integrated in a non-linear competitive inhibition equation using the GraphPad software (Prism) for the *K*_i_ calculation. Endpoint NA activity was measured by MU-NANA assay using a standardized HA titer of viruses at 37°C for 1 hr.

### Affinity and specificity for the sialic acid receptors

The HA affinity assays were performed as previously described [11], [20]. Briefly, viral supernatants were coated onto 96-well plates overnight at room temperature. Biotynilated sialylglycopolymers Neu5Acα2-3Galβ1-4Glc-PAA-Biotin (3′SL) and Neu5Acα2-6Galβ1-4GlcNAc-PAA-Biotin (6′SLN) (Lectinity) were serially diluted in 10 nM of zanamivir and incubated for 2 h at 4°C. After washing, peroxidase-labeled anti-biotin antibodies (Euromedex) were added. TMB (KPL) was used as a substrate and absorbance was recorded at 450 nm (Microplate reader UMV340 Asys, Bioserv). The dissociation constant (Kd) was calculated with a non-linear one site specific binding equation using the GraphPad software.

### Mice challenge

Anesthetized mice were inoculated intra-nasally with virus (n = 3 per group; 20 µl of 10^6^, 10^5^, 10^4^, 10^3^ or 10^2^ TCID_50_). For determination of the mouse infectious dose (MID_50_), mice were euthanized at 3 days post infection (d.p.i.). Lungs were collected and stored at −70°C. The frozen tissues were thawed, homogenized in 0.5 ml of PBS, disrupted in a Tissue Lyser (Qiagen) and then clarified by centrifugation (2000 *g*) at 4°C. Supernatants were titrated by end point dilution using the MDCK cells and confirmed using the hemagglutination assays.

### Data collection

All data were saved using Tomuss, an interactive web application which allows multiple concurrent users to edit data tables.

## Results

### Whole-genome analysis of influenza sequences from clinical isolates

We performed sequencing using early passages (<3) of viral isolates (Table 2–4), and classified the viruses into genetic groups according to the ECDC technical document of August/September 2011 [21]. Viruses isolated in 2009 (Ste09G and Lyon09D) were phylogenetically related to genetic group 1 that included the reference strain A/California/07/2009. Those isolated in 2010, (Ste10D, Lim10G, Lyon10N) were phylogenetically related to the genetic group 3, characterized by the mutations A134T and S183P in HA. Lyon11E and Reu10G were phylogenetically related to genetic group 6, characterized by the mutations D97N and S185T in HA. The differences between the coding sequences and among each group ranged from 11 to 29 amino acids, respectively.

**Table 2:** Amino acid divergence of the HA and NA of H1N1pdm viruses.

Protein	HA												NA							
AA Positions	32	56	97	119	134	183	185	203	222^a^	374	441	451	13	15	93	106	241	248	369	394
Antigenic sites^b^							Sb^b^	Ca1^b^	Ca2^b^											
Lyon09D	L	N	D	K	A	S	S	S	**D**	E	N	S	V	M	P	V	V	N	N	V
Ste10D	.	.	.	.	T	P	.	T	**D**	.	K	.	.	I	.	I	.	D	.	I
Ste09G	I	.	.	.	.	.	.	.	**G**	.	.	.	.	.	.	.	.	.	.	.
Lim10G	.	.	.	.	T	P	.	T	**G**	.	K	.	.	.	.	I	I	D	.	I
Reu10G	.	.	N	N	.	.	T	T	**G**	K	.	N	I	.	S	I	I	.	K	.
Lyon10N	.	.	.	.	T	P	.	T	**N**	.	K	.	.	.	.	I	.	D	.	I
Lyon11E	.	S	N	.	.	.	T	T	**E**	K	.	N	.	.	.	I	I	D	K	.

a Residues forming the receptor binding pocket are in bold.

b Amino acids in antigenic sites, Sb, Ca1 and Ca2.

**Table 3:** Amino acid divergence of the PB2, PB1 and PA of H1N1pdm viruses.

Protein	PB2										PB1								PA			
AA Positions	90	184	214	255	344	354	511	526	574	588	176	211	257	353	566	645	648	652	85	321	479	581
Lyon09D	M	A	K	V	V	I	V	R	K	T	K	R	T	K	T	V	A	A	I	N	D	L
Ste10D	V	.	R	I	.	.	I	K	.	I	.	K	.	.	.	.	G	V	.	.	E	M
Ste09G	L	.	.	.		.	.	K	.	.	.	.	.	R	S	.	.	.	.	.	.	.
Lim10G	V	.	R	I	.	.	I	K	R	I	.	K	A	.	.	.	.	V	.	.	E	M
Reu10G	Nd^a^	nd	nd	nd	nd	nd	nd	nd	nd	nd	.	.	.	.	.	I			V	K	.	M
Lyon10N	V	T	R	I	.	.	I	K	.	I	.	K	.	.	.	.	.	V	.	.	E	M
Lyon11E	M	.	.	.	M	L	.	K	.	.	R	.	.	.	.	.	.	.	.	K	.	M

a nd: not done.

**Table 4:** Amino acid divergence of the NP, M1, NS1, NS2 and PA-X of H1N1pdm viruses.

Protein	NP							M1		NS1							NS2	PA-X		
AA Positions	16	100	133	270	365	373	433	80	133	55	111	114	123	145	162	189	32	85	215	220
Lyon09D	G	V	I	V	I	I	S	V	N	E	I	P	I	I	P	G	V	I	T	R
Ste10D	.	I	L	.	.	T	N	.	S	Q	L	.	V	.	.	.	.	.	.	.
Ste09G	D	.	L	F	.	T	N	.	.	L	F	.	T	.	.	.	.	.		.
Lim10G	.	I	L	.	.	T	N	.	S	Q	.	.	V	.	.	.	.	.	.	.
Reu10G	.	I	L	.	.	T	.	I	.	.	.	.	V	V	.	.	.	V	.	Q
Lyon10N	.	I	L	.	.	T	N	.	S	Q	.	.	V	.	S	.	.	.	.	.
Lyon11E	.	I	L	.	S	T	N	I	.	.	.	T	V	.	.	D	I	.	I	Q

All seven isolates harbored P83S and I321V substitutions in the HA. The HA sequences of Lyon09D and Ste09G differed by D222G and L32I, while those of Ste10D, Lim10G and Lyon10N were strictly identical except for the 222 polymorphism.

The NA protein sequences of Lyon09D and Ste09G were identical and apart from M15I, so too were those of Ste10D, Lim10G and Lyon10N. All viruses except for Ste09G harbored the NA substitution V106I. Lyon09D, Reu10G and Ste10D also harbored the NA N248D substitution. We found no mutation associated with resistance for NA (Table 2, Table 3).

One to eight mutations concerned NP, PB1 and PB2 and none to three substitutions NS1 and NS2. In contrast, the matrix protein (M) had one substitution (V80I or N133I). Overall, none of the substitutions detected has been reported to be associated with increased virulence. We assume that they reflected polymorphism.

### Hemagglutinin properties

Using a panel of antisera raised against H1 influenza vaccine reference viruses, we determined the antigenic properties of the seven viruses by HI assays (Table S1). The HA of all seven viruses was antigenically related to that of the H1N1pdm reference strain. Therefore the substitutions observed in the HA proteins did not induce any antigenic drift.

To assess the changes in HA receptor binding induced by the 222 polymorphism, we evaluated the HA binding profiles of the seven isolates in terms of binding affinity (Kd) and binding specificity for SAα-2,6 (Neu5Acα2-6Galβ1-4GlcNAc-PAA-Biotin) and SAα-2,3 (Neu5Acα2-3Galβ1-4Glc-PAA-Biotin) sialylglycopolymers (Table 5). For all of the viruses, the intensity of binding affinity to SAα-2,6 was higher than that to SAα-2,3 (mean Kd values of 0.15 µM and 1.19 µM, respectively). However, we noted marked differences amongst the the three G222 viruses (*i.e* Reu10G, Lim10G, Ste09G), the two D222 viruses (*i.e* Lyon09D, Ste10D) and the two N and E222 viruses (*i.e* Lyon10N, Lyon11E), with regards binding to SAα-2,3 and SAα-2,6. The G222 viruses bound SAα-2,3 (mean Kd value of 0.79 µM) with higher intensity and SAα-2,6 (mean Kd value 0.29 µM) with lower intensity compared with the two D222 viruses (mean Kd values to SAα-2,3 and SAα-2,6 of 2.57 µM and 0.06 µM, respectively). Finally, N and E222 viruses bound to both SAα-2,3 (mean Kd value of 0.41 µM) and SAα-2,6 (mean Kd value of 0.02 µM) with higher intensity compared with the other five viruses (Table 5).

**Table 5:** HA and NA properties of H1N1pdm HA222 variants.

Strain	HA 222	HA affinity		NA properties			
		3′SL	6′SLN	Km^b^	NA activity	Oseltamivir	Zanamivir
		Kd^a^ (µM)		(µM)	(nmol/h/10^6^ viruses)	IC50^c^ (nM)	IC50^c^ (nM)
Lyon09D	D	1.61	0.07	34.7	9.1	0.67	1.46
Ste10D	D	3.52	0.05	23.8	23.0	0.47	0.40
*Mean D222 viruses*		*2.57±0.96*	*0.06±0.01*	*29.3±5.5*	*16.0±6.9*	*0.57±0.1*	*0.93±0.5*
Ste09G	G	0.63	0.11	40.4	1.8	0.51	0.96
Lim10G	G	0.74	0.36	31.5	1.4	0.36	0.38
Reu10G	G	0.99	0.39	29.8	1.7	0.53	1.76
*Mean G222 viruses*		*0.79±0.11*	*0.29±0.09*	*33.9±3.3*	*1.7±0.1*	*0.47±0.1*	*1.0±0.4*
Lyon10N	N	0.39	0.01	34.3	4.5	0.24	0.56
Lyon11E	E	0.42	0.03	42.6	4.2	0.50	0.68
*Mean E/N 222 viruses*		*0.41±0.02*	*0.02±0.01*	*38.5±4.1*	*4.4±0.1*	*0.37±0.1*	*0.62±0.1*

a Dissociation constant Kd of the viruses for 6′SLN sialylglycopolymer (Neu5Acα2-6Galβ1-4GlcNAc-PAA-Biotin) and 3′SL sialylglycopolymer (Neu5Acα2-3Galβ1-4Glc-PAA-Biotin) were calculated using the non-linear one site specific binding equation using the GraphPad software. The values are the mean of two assays.

b Michaelis Menten constants (Km) were determined with MU-NANA ranging from 10 to 200 µM. Results are given as the mean Km values calculated using values derived from three separate assays.

c Neuraminidase inhibition assays (IC50) were performed on cell supernatants with MU-NANA as a substrate at a final concentration of 50 µM. Values were obtained from duplicate samples.

Altogether, these results indicate that the HA D222G substitution induced a shift in the binding profile by increasing the affinity for SAα-2,3 and decreasing the affinity for SAα-2,6, while the presence of N or E at position 222 increased the binding affinity for both SAα-2,3 and SAα-2,6 substrates.

### Neuraminidase properties

All viruses were sensitive to both zanamivir and oseltamivir carboxylate with similar low IC_50_ (Table 5) and inhibition constants (*K*_i_) (data not shown). This is consistent with our sequence analysis showing a lack of the amino acid changes known to confer resistance in the NA sequence of the seven isolates [22].

Kinetic analyses of the sialidase activity of NA were performed using the MUNANA fluorogenic substrate, a non-specific link substrate of sialidase. NA efficiency was determined using the Michaelis-Menten constant (*K*_m_), which reflects the affinity for the substrate, and the V_max_, which reflects the activity of the enzyme (Table 5). The *K*_m_ value for the MUNANA substrate of Ste10D (23.8 µM) was reduced compared with those measured for the other viruses (mean *K*_m_ 35.5 µM). Two viruses showed a high *K*_m_ value above 40 µM: Ste09D (40.4 µM) and Lyon11E (42.6 µM). The mean NA activity of the D222 HA viruses (16.0 nmol/h/106 viruses) was higher than the mean obtained for G222 HA viruses (1.7 nmol/h/106 viruses) and E/N222 viruses (4.4 nmol/h/106 viruses). Despite differing from the Lyon09D and Ste09G NA only by the M15I substitution, Ste10D NA activity was two to ten-fold higher (Table 5).

### HA-NA balance

**Figure 1:**
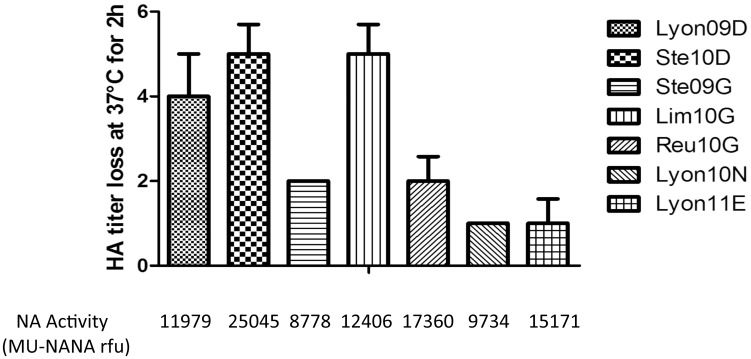
Quantitative assessment of HA binding and the associated NA activity of H1N1pdm HA 222 variants. Initial HA titers were measured using guinea pig red blood cells after 75 min incubation at 4°C and final HA titers were determined after 120 min incubation at 37°C as described in Methods. The end point NA activity was measured on 16 UHA viruses diluted ten-fold and incubated with MU-NANA at 37°C for 1 hr.

We then used guinea pig red blood cells to provide a quantitative assessment of the binding and release of the seven viruses. The HA titer assay was first performed at 4°C in order to inhibit NA activity and then at 37°C to activate NA (Figure 1). HA titers were reduced by four to five fold for the Lyon09D, Ste10D and Lim10G viruses, by two fold for Ste09G and Reu10G viruses and by one fold for Lyon11E and Lyon10N viruses. These data suggest that the HA-NA balance of Lyon09D, Ste10D and Lim10G differs from that of Lyon11E and Lyon10N.

**Figure 2:**
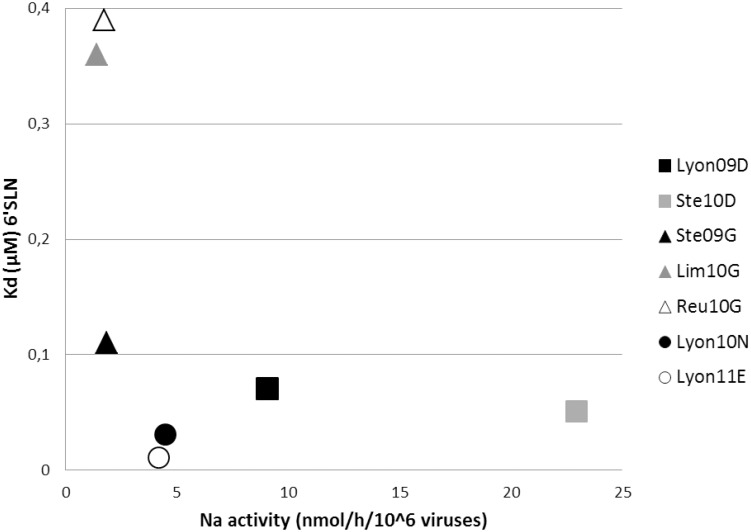
Functional HA-NA balance of H1N1pdm HA 222 variants. The relationship between the activity of HA and NA was assessed by plotting the quantitative results from the HA binding affinity assay with the NA activity.

We then assessed the relationship between HA and NA activities by plotting the quantitative results from the HA binding affinity (Kd) assay with the NA activity assay (nmol/h/10^6^ viruses) (Figure 2) [23]. Figure 2 highlights two major features: firstly, Lim10G, Reu10G and Ste10D viruses presented the same HA-NA balance. Yet while Lim10G and Reu10G viruses displayed a low HA binding intensity to sialic acids and a low NA activity, the Ste10D strain conversely displayed a high HA binding intensity to sialic acids and a high NA activity. Secondly, the Lyon11E and Lyon10N strains deviated from such balance with a high HA binding affinity and a low NA activity.

### Growth properties in vitro and in vivo

**Figure 3:**
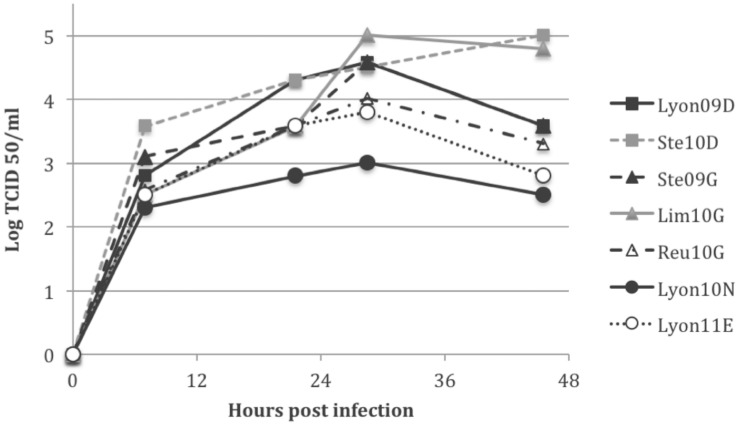
Replication kinetics of H1N1pdm viruses in MDCK-SIAT cells at a MOI of 0.001.

The isolates displayed considerable differences in terms of viral growth and final titers *in vitro* in MDCK SIAT-1 cells, which express high levels of α-2,6-linked sialic acid receptors (Figure 3). Indeed, while Ste10D and Lim10G viruses replicated efficiently (10^5^ TCID50/ml at 48 hrs p.i.) in the MDCK SIAT-1 cells, Lyon11E and Lyon10N viruses displayed a more than two log_10_ lower efficiency (<10^3^ TCID50/ml at 48 hrs p.i.). These findings confirm that, at least *in vitro*, an imbalance between the activities of HA and NA impairs the replicative capacities (i.e. fitness) of the viruses with N or E at position 222 of HA.

The mouse model has proven to be a useful tool to analyze influenza viruses associated with human infection [24]. To evaluate the effect of both receptor specificity and HA-NA balance on the replicative ability of the viruses studied *in vivo*, the MID50 and virulence were determined in mice (Table 6). All H1N1pdm viruses but one exhibited an MID50 between 0.5×10^3^ and 1.6×10^3^ and replicated to high titers (>10^6.9^ TCID50/g lung) in the lungs of infected animals, indicating a high degree of infectivity in this model [20] [25]. The Ste10D strain had a ten-fold higher MID50 (15×10^3^ MID50) compared with the Lyon09D or Lim10G strains which was corroborated by low titers (10^5.5^ TCID50/g lung) in the lungs of infected animals indicating a low infectivity of this strain in BALB/C mice. Taken together, these results indicate that N or E at position 222 of HA alters the replication of influenza in MDCK SIAT1 cells but not in mice. Moreover, the fitness of the Ste10D strain was altered in mice but not in MDCK SIAT1 cells.

**Table 6:** Infectivity and virulence of H1N1pdm HA 222 variants in mice.

Virus	Mice Infection	
	10^3^ MID50^a^	Log TCID50/g lung ± SD^b^
Lyon09D	1.6	7.3±0.2
Ste10D	15.8	5.5±0.9
Ste09G	0.5	6.9±0.5
Lim10G	1.4	7.5±0.1
Reu10G	0.9	7.4±0.5
Lyon10N	0.5	7.3±0.2
Lyon11E	1.0	6.9±0.2

Three mice were intranasally infected with 10^5^ TCID50 and were euthanized at 3 d.p.i. The results are given as the mean value of three infected mice.

a Mouse infectious dose 50% (MID50) were expressed as the TCID50/20 µl required for one MID50. Group of three mice were infected with 10^6^,10^5^, 10^4^, 10^3^ or 10^2^ TCID50 and viruses were titrated in the lungs.

b Viral titers in the lungs of inoculated mice (n = 3) with 10^5^ TCID50 were determined at 3 d.p.i. SD: Standard Deviation.

## Discussion

Our work was designed to assess the impact of amino acid substitutions at HA 222 on HA receptor binding properties, on the HA-NA functional balance and on viral growth and virulence. We selected seven clinical isolates that reflected the HA 222 polymorphism. Firstly, we analyzed SAα-2,6 and SAα-2,3 HA receptor binding properties. Our results confirmed that HA G222 resulted in a higher binding intensity to SAα-2,3 and a lower binding intensity to SAα-2,6, as previously reported [8]. Moreover, we observed that HA N222 and E222 displayed an increased binding intensity for both SAα-2,3 and SAα-2,6. Secondly, we analyzed the enzymatic properties of NA and assessed the functional HA-NA balance. The mean NA activity of D222 HA viruses was higher than that of either the G222 HA viruses or E/N222 viruses. Accordingly, Lyon11E and Lyon10N viruses displayed a strong HA binding unbalanced by a weak NA activity. Therefore the HA-NA balance of Lyon11E and Lyon10N deviated from that of Lim10G, Reu10G, and Ste10D. We further evaluated potential differences in the HA-NA balance among viruses using the hemagglutination assay. In agreement with previous data, Lyon11E and Lyon10N viruses failed to elute red blood cells after 2 hrs whereas HA D222 and one of the HA G222 (Lim10G) viruses eluted efficiently [23]. Lyon11E and Lyon10N did not replicate efficiently *in vitro* in MDCK SIAT-1 cells despite a higher binding intensity to SAα-2,6.

These findings confirm that, at least *in vitro*, an imbalance between the activities of HA and NA significantly impairs the replicative capacities (*i.e.* fitness) of the viruses. The HA D222G/E/N substitution increases the intensity of binding for SAα-2,6 and alters the HA-NA balance which may decrease viral fitness in a SAα-2,6 environment. This may explain why D222G and also D222E/N viruses are not able to be transmitted efficiently and are isolated only sporadically.

We observed some discrepancies between the HA binding affinity, NA activity and hemagglutination assays. Indeed, while Reu10G presented a similar functional balance to Lim10G, it's HA titer was reduced by two-fold compared to five-fold for Lim10G. This may be explained by the difference in sialic acid used in the HA binding affinity, NA activity and hemagglutination assays.

The use of human influenza isolates is one limitation of this study. Viruses not only exhibit a polymorphism on the segments encoding the surface glycoproteins, but also in the internal segments. While full genome sequencing did allow us to draw some conclusions with regards the lack of mutations known to confer specific properties found in the different segments of these isolates, we cannot exclude that some slight genetic differences may contribute towards a change in fitness *in vivo*. The limited number of isolated strains did not allow us to conclude on the potential impact of the D222G or HA-NA balance in human pathogenicity.

While acquisition of a lethal phenotype has been shown in viruses affecting mice following the acquisition of key mutations, one of which is D222G [8], we found no such differences in human influenza viruses and D222G did not appear as a main virulence factor in mice [20]. The use of the mouse model revealed a mouse infectious dose (MID50) ranging from 0.5×10^3^ to 1.6×10^3^ but no significant differences in the replication rate in the lungs for all viruses, except for the Ste10D virus. In accordance with other groups [24], [25], [26], none of the viruses we studied with our mouse model showed an increase in virulence. The low fitness of the Ste10D virus may be due to the predominance of SAα-2,3 in mice [27] and a low HA binding affinity for the SAα-2,3 substrate. On the other hand, although Lyon11E and Lyon10N viruses replicated at low levels in the SAα-2,6 system (i.e. MDCK SIAT-1 cells), they had a low MID50 and replicated to high titers in the lungs of the mice. These results indicate that mice are an effective *in vivo* model to assess SAα-2,3 HA binding affinity, but not for assessing the receptor preference or the HA-NA balance as previously reported [26]. Other properties that affect the virulence of influenza viruses in mice, such as the complexity of the sialic acid species in the mouse respiratory tract, could also explain these discrepancies.

The NA activity of the Ste10D virus was higher compared with the other viruses studied, yet differed from the Lyon09D and Ste09G NA by only the M15I substitution. This position is located in the transmembrane domain and is not likely to affect the structure of the NA pocket. Such a polymorphism has already been observed [28] but not analyzed functionally. This mutation may modify monomers in terms of their anchorage or interactions, with repercussions on NA expression on the virus surface.

This is the first report, to our knowledge, that has studied the functional balance of HA-NA in human strains regarding the impact of the D222G/N/E substitution on SAα-2,6 HA binding. These results confirm the importance of an optimal balance between HA affinity and NA activity for human influenza viruses with regards viral fitness [29]. It appears therefore crucial to take into account both HA receptor binding and NA receptor cleavage in terms of the HA-NA balance in the context of a mutation affecting HA binding properties, by using enzymatic and biological tools.

## Supporting Information

Table S1 **Hemagglutinin antigenic properties determined by the hemagglutination inhibition assay.**
(DOCX)

Table S2 **Primers for the whole genome sequencing of H1N1pdm viruses.**
(DOCX)

Table S3 **GenBank accession numbers for the seven H1N1pdm09 isolates.**
(DOCX)
