# Supplementary material for: Functional Balance between the Hemagglutinin and Neuraminidase of Influenza A(H1N1)pdm09 HA D222 Variants
Source: PLoS One. 2014 Aug 13;9(8):e104009. doi: 10.1371/journal.pone.0104009 (PMC4131921; doi:10.1371/journal.pone.0104009)
Supplement: Table S1 — Hemagglutinin antigenic properties determined by the hemagglutination inhibition assay. (DOCX) [file pone.0104009.s001.docx]

**Table S1** Hemagglutinin antigenic properties determined by the hemagglutination inhibition assay

|  |  |  |  |  |  |
| --- | --- | --- | --- | --- | --- |
|  | A/Brisbane/59/2007 | A/New Jersey/8/1976 | A/Auckland/3/2009 | A/California/7/2009 | A/Lyon/969/2009 |
| Rabbit (L) and Ferret (F) sera | R772 | F221 | F/C4/17/09 | F/C4/16/09 | F773 |
| Source of sera | National Influenza Centre (South of France) | National Influenza Centre (South of France) | WHO Influenza Centre at NIMR London | WHO Influenza Centre at NIMR London | National Influenza Centre (South of France) |
| **Reference strains** |  |  |  |  |  |
| A/Brisbane/59/2007 | **10240**^a^ | <^b^ | < | < | < |
| A/New Jersey/8/1976 | 320 | **640** | 320 | 320 | 640 |
| A/Auckland/3/2009 | 160 | 320 | **5120** | 2560 | 2560 |
| A/Califormia/7/2009 | < | < | 320 | **320** | 640 |
| A/Lyon/969/2009 | 80 | 320 | /^c^ | 1280 | **1280** |
|  |  |  |  |  |  |
| **Isolates** |  |  |  |  |  |
| A/StEtienne/1691/2009 | < | 80 | / | 320 | / |
| A/Reunion/803/2010 | < | 80 | 1280 | 640 | / |
| A/Lyon/01.012/2011 | < | 160 | 1280 | / | / |
| A/StEtienne/1139/2010 | < | 160 | / | 640 | / |
| A/Limoges/1159/2010 | < | 160 | / | 640 | / |
| A/Lyon/52.016/2010 | < | 80 | 640 | / | / |

^a^ Homologous reaction are in bold.

^b^ < : Titer less than 10.

^c^ / : not determined.
