# Supplementary material for: Functional Balance between the Hemagglutinin and Neuraminidase of Influenza A(H1N1)pdm09 HA D222 Variants
Source: PLoS One. 2014 Aug 13;9(8):e104009. doi: 10.1371/journal.pone.0104009 (PMC4131921; doi:10.1371/journal.pone.0104009)
Supplement: Table S2 — Primers for the whole genome sequencing of H1N1pdm viruses. (DOCX) [file pone.0104009.s002.docx]

**Table S2** : Primers for the whole genome sequencing of H1N1pdm viruses

WHO refers to primers designed by the WHO (<http://www.who.int/csr/resources/publications/swineflu/sequencing_primers/en/>)

Virpath refers to primers designed by the laboratory

| Segment | Forward Primer | Sequence | Position | Reverse Primer | Sequence | Position |
| --- | --- | --- | --- | --- | --- | --- |
| PB2 | *WHO*: PB2_FOR-1 | TGTAAAACGACGGCCAGTCTCGAGCAAAAGCAGGTCAA | 1-38 | *WHO :*PB2_REV-816 | CAGGAAACAGCTATGACCGCTTTGRTCAAYATCRTCATT | 777-816 |
|  | *WHO*: PB2_FOR-713 | TGTAAAACGACGGCCAGTCAAGCAGTRTRTACATTGAAGT | 713-753 | *WHO*: PB2_REV-1509 | CAGGAAACAGCTATGACCGGAGTATTCATCYACACCCAT | 1470-1509 |
|  | *WHO*: PB2_FOR-1447 | TGTAAAACGACGGCCAGTCCAAGYACMGAGATGTCAATGAGA | 1447-1489 | *WHO*: PB2_REV-2341 | CAGGAAACAGCTATGACCTAGTAGAAACAAGGTCGTT | 2304-2341 |
| PB1 | WHO: PB1_FOR-22 | TGTAAAACGACGGCCAGTAGCAAAAGCAGGCAAACCAT | 22-60 | WHO: PB1_REV-843 | CAGGAAACAGCTATGACCGTTCAAGCTTTTCRCAWATG | 805-843 |
|  | *WHO*: PB1_FOR-711 | TGTAAAACGACGGCCAGTTGAACACRATGACCAARGA | 711-748 | *WHO*: PB1_REV-1566 | CAGGAAACAGCTATGACCAGCTCCATGCTRAAATTRGC | 1528-1566 |
|  | *WHO*: PB1_FOR-1489 | TGTAAAACGACGGCCAGTATGAGYAAAAAGAAGTCYTA | 1489-1527 | *WHO*: PB1_REV-2321 | CAGGAAACAGCTATGACCAGTAGAAACAAGGCATTT | 2285-2321 |
| PA | WHO: PA_FOR-0 | TGTAAAACGACGGCCAGTAGCAAAAGCAGGTACTGAT | 0-37 | WHO : PA_REV-989 | CAGGAAACAGCTATGACCGGTTCTTTCCATCCAAAGAATGTT | 947-989 |
|  | *WHO*: PA_FOR-894 | TGTAAAACGACGGCCAGTAAATTRAGCATTGARGAYCCG | 894-933 | *WHO*: PA_REV-1662 | CAGGAAACAGCTATGACCTCWAGTCTYGGGTCAGTGAG | 1624-1662 |
|  | *WHO*: PA_FOR-1444 | TGTAAAACGACGGCCAGTAATGCATCCTGTGCAGCAATGGA | 1444-1485 | *WHO*: PA_REV-2233 | CAGGAAACAGCTATGACCAGTAGAAACAAGGTACCTTTT | 2194-2233 |
| HA | WHO : HA-FOR-1 | TGTAAAACGACGGCCAGTATACGACTAGCAAAAGCAGGGG | 1-40 | WHO : HA-REV-943 | CAGGAAACAGCTATGACCGAAAKGGGAGRCTGGTGTTTA | 904-943 |
|  | *WHO*: HA-FOR-736 | TGTAAAACGACGGCCAGTAGRATGRACTATTACTGGAC | 736-774 | *WHO*: HA-REV-1778 | CAGGAAACAGCTATGACCGTGTCAGTAGAAACAAGGGTGTTT | 1736-1778 |
| NP | Virpath : NP-For-Esp3I | TTCGTCTCAGGGAGCAAAAGCAGGGTAGAT | 1-30 | Virpath : NP-Rev-Esp3I | ATATCGTCTCGTATTAGTAGAAACAAGGGTATTTTT | 1529-1565 |
| NA | WHO : NA-FOR-0 | TGTAAAACGACGGCCAGTAGCAAAAGCAGGAGT | 0-33 | WHO : NA-REV-740 | CAGGAAACAGCTATGACCGGRCCATCGGTCATTATG | 704-740 |
|  | *WHO*: NA-FOR-726 | TGTAAAACGACGGCCAGTAATGGRCARGCCTCRTACAA | 726-764 | *WHO*: NA-REV-1452 | CAGGAAACAGCTATGACCAGTAGAAACAAGGAG | 1419-1452 |
| M | Virpath : M_For-Esp3I | TATTCGTCTCAGGGAGCAAAAGCAGGTAG | 1-29 | Virpath M_Rev-Esp3I | ATATCGTCTCGTATTAGTAGAAACAAGGTAGTTTTT | 991-1027 |
| NS | Virpath NS_For-Esp3I | TATTCGTCTCAGGGAGCAAAAGCAGGGTG | 1-29 | Virpath NS_Rev-Esp3I | ATATCGTCTCGTATTAGTAGAAACAAGGGTGTTTT | 855-890 |
