# Supplementary material for: Functional Balance between the Hemagglutinin and Neuraminidase of Influenza A(H1N1)pdm09 HA D222 Variants
Source: PLoS One. 2014 Aug 13;9(8):e104009. doi: 10.1371/journal.pone.0104009 (PMC4131921; doi:10.1371/journal.pone.0104009)
Supplement: Table S3 — GenBank accession numbers for the seven H1N1pdm09 isolates. (DOCX) [file pone.0104009.s003.docx]

**Table S3** GenBank accession numbers for the seven H1N1pdm09 isolates

| strains H1N1 | Short name | HA 222 polymorphism | segment 1 PB2 | segment 2 PB1 | segment 3 PA | segment 4 HA | segment 5 NP | segment 6 NA | segment 7 M | segment 8 NS |
| --- | --- | --- | --- | --- | --- | --- | --- | --- | --- | --- |
| A/Lyon/969/2009 | Lyon09D | D | KC800977 | KC800978 | KC800979 | JF429402 | KC800980 | JF429403 | KC800981 | KC800982 |
| A/StEtienne/1691/2009 | Ste09G | G | KF897769 | KF897770 | KF897771 | JF429396 | KF897772 | JF429401 | KF897773 | KF897774 |
| A/LaRéunion/803/2010 | Reu10G | G | Not done | KF897775 | KF897776 | KF897777 | KF897778 | KF897779 | KF897780 | KF897781 |
| A/Lyon/52.16/2010 | Ste10D | N | KF897782 | KF897783 | KF897784 | KF897785 | KF897786 | KF897787 | KF897788 | KF897789 |
| A/StEtienne/1139/2010 | Lim10G | D | KF897790 | KF897791 | KF897792 | KF897793 | KF897794 | KF897795 | KF897796 | KF897797 |
| A/Limoges/1159/2010 | Lyon10N | G | KF897798 | KF897799 | KF897800 | KF897801 | KF897802 | KF897803 | KF897804 | KF897805 |
| A/Lyon/1.12/2011 | Lyon11E | E | KF897806 | KF897807 | KF897808 | KF897809 | KF897810 | KF897811 | KF897812 | KF897813 |
